# Multimodal immersive trail making-virtual reality paradigm to study cognitive-motor interactions

**DOI:** 10.1186/s12984-021-00849-9

**Published:** 2021-05-17

**Authors:** Meir Plotnik, Oran Ben-Gal, Glen M. Doniger, Amihai Gottlieb, Yotam Bahat, Maya Cohen, Shani Kimel-Naor, Gabi Zeilig, Michal Schnaider Beeri

**Affiliations:** 1 Center of Advanced Technologies in Rehabilitation, Sheba Medical Center, Ramat Gan, Israel; 2 Department of Physiology and Pharmacology, Sackler Faculty of Medicine, Tel Aviv University, Tel Aviv, Israel; 3 Sagol School of Neuroscience, Tel Aviv University, Tel Aviv, Israel; 4 Joseph Sagol Neuroscience Center, Sheba Medical Center, Ramat Gan, Israel; 5 Department of Neurological Rehabilitation, Sheba Medical Center, Ramat Gan, Israel; 6 Deartment of Rehabilitation, Sackler Faculty of Medicine, Tel Aviv University, Tel Aviv, Israel; 7 Department of Psychiatry, The Icahn School of Medicine At Mount Sinai, New York, NY USA

**Keywords:** Executive functions, Cognitive-motor interactions, Construct validity, Divided attention, Neuropsychological testing, Virtual reality

## Abstract

**Background:**

Neuropsychological tests of executive function have limited real-world predictive and functional relevance. An emerging solution for this limitation is to adapt the tests for implementation in virtual reality (VR). We thus developed two VR-based versions of the classic Color-Trails Test (CTT), a well-validated pencil-and-paper executive function test assessing sustained (Trails A) and divided (Trails B) attention—one for a large-scale VR system (DOME-CTT) and the other for a portable head-mount display VR system (HMD-CTT). We then evaluated construct validity, test–retest reliability, and age-related discriminant validity of the VR-based versions and explored effects on motor function.

**Methods:**

Healthy adults (*n* = 147) in three age groups (young: *n* = 50; middle-aged: *n* = 80; older: *n* = 17) participated. All participants were administered the original CTT, some completing the DOME-CTT (14 young, 29 middle-aged) and the rest completing the HMD-CTT. Primary outcomes were Trails A and B completion times (t_A_, t_B_). Spatiotemporal characteristics of upper-limb reaching movements during VR test performance were reconstructed from motion capture data. Statistics included correlations and repeated measures analysis of variance.

**Results:**

Construct validity was substantiated by moderate correlations between the’gold standard’ pencil-and-paper CTT and the VR adaptations (DOME-CTT: t_A_ 0.58, t_B_ 0.71; HMD-CTT: t_A_ 0.62, t_B_ 0.69). VR versions showed relatively high test–retest reliability (intraclass correlation; VR: t_A_ 0.60–0.75, t_B_ 0.59–0.89; original: t_A_ 0.75–0.85, t_B_ 0.77–0.80) and discriminant validity (area under the curve; VR: t_A_ 0.70–0.92, t_B_ 0.71–0.92; original: t_A_ 0.73–0.95, t_B_ 0.77–0.95). VR completion times were longer than for the original pencil-and-paper test; completion times were longer with advanced age. Compared with Trails A, Trails B target-to-target VR hand trajectories were characterized by delayed, more erratic acceleration and deceleration, consistent with the greater executive function demands of divided vs. sustained attention; acceleration onset later for older participants.

**Conclusions:**

The present study demonstrates the feasibility and validity of converting a neuropsychological test from two-dimensional pencil-and-paper to three-dimensional VR-based format while preserving core neuropsychological task features. Findings on the spatiotemporal morphology of motor planning/execution during the cognitive tasks may lead to multimodal analysis methods that enrich the ecological validity of VR-based neuropsychological testing, representing a novel paradigm for studying cognitive-motor interactions.

**Supplementary Information:**

The online version contains supplementary material available at 10.1186/s12984-021-00849-9.

## Background

The term “executive functions” is an umbrella term for a wide range of cognitive processes and behavioral competencies necessary for the cognitive control of behavior including problem solving, planning, sequencing, sustained attention, utilization of feedback, and multitasking [1]. Neuropsychological tests of executive functions aim to assess these processes [2]. Accordingly, performance on these tests is assumed indicative of executive functioning in everyday living [3]. One of the limitations of these tests relates to their low ‘ecological validity’, namely the uncertainty about how closely they reflect capacity of executive function in real life [4–6]. In this regard, Burgess et al. [7] has claimed that “the majority of neuropsychological assessments currently in use were developed to assess 'cognitive constructs' without regard for their ability to predict 'functional behavior'."

### Neuropsychological assessment in virtual reality (VR)

Early discussions of ecological validity in neuropsychology emphasized that the technologies available at that time could not replicate the setting in which the behavior of interest actually occurs [8]. Furthermore, currently, most neuropsychological assessments still use outdated methods (e.g., pencil-and-paper administration; static stimuli) that have yet to be validated with respect to real-world functioning [9].

To overcome this limitation, testing participants in real word situations (e.g., the Multiple Errands Test [MET] [10]) has been considered an ecologically valid and advantageous alternative to traditional tests [11]. However, this approach is logistically challenging, requiring travel to a naturalistic testing site [12].

In an attempt to overcome this logistical hurdle, the Virtual Errands Test (VET) was devised by McGeorge et al. [13] as an adaptation of the MET for VR-based administration. Still, this test, and similar VR variants, are limited in their ability to distinguish between healthy and clinical cohorts (see [11] for a review) and to yield performance on the virtual tasks similar to performance in the real world (e.g., [14, 15]). Further, most VR-based tests like VET involve presenting a simulated VR environment on a standard computer screen (e.g., Elkind et al. [16]), which may lead to a non-immersive experience, thus paradoxically compromising rather than enhancing ecological validity.

Notably, VR-based tests simulating shopping tasks for the assessment of executive function have demonstrated good ecological validity [17, 18]. However, the approach of adapting executive function testing for the VR environment has not been widely accepted in both research and clinical contexts.

### Research rationale

Critically, we posit that the concept of 'ecological validity' is not merely related to the type of task performed and its relevance to daily living. In general, each response on a cognitive task involves interactions with sensory and motor functions, first to determine the required behavioral response and then to plan and execute it. These processes cannot be distinguished and examined with traditional pencil-and-paper testing or even with computerized testing platforms.

Thus, as a first step, we aim to develop VR neuropsychological tests by adapting well-validated traditional neuropsychological tests that measure particular cognitive constructs. These adaptations will enhance ecological validity by including multi-multimodal (e.g., cognitive-sensory-motor) interactions, facilitating measurement of cognitive function in a manner more relevant to to the interaction among multiple functions characteristic of everyday activities [19–24]. Specifically, the VR technology we employ allows for collection of quantitative three-dimensional kinematic data (unavailable for traditional neuropsychological tests) that tracks motion in space and may improve our ability to define and discriminate among levels of performance.

### The Color Trails Test (CTT)

The Trail Making Test (TMT) [25, 26] is among the most popular pencil-and-paper tests of executive function, attention and processing speed in research and clinical neuropsychological assessment. The Color Trails Test (CTT) is a culture-fair variant of the TMT. In *Trails A* the participant draws lines to sequentially connect circles numbered 1–25 (odd-numbered circles are pink; even-numbered circles are yellow). In *Trials B* the participant alternates between circles of two different colors (i.e., 1-pink, 2-yellow, 3-pink, 4-yellow, etc.) [27]. Scoring is based on the time needed to complete the tasks, with shorter time reflecting better performance. It has been proposed that *Trails A* assesses sustained visual attention involving perceptual tracking and simple sequencing, while Trails B more directly assesses executive function processes, including divided attention, simultaneous alternating and sequencing [27, 28].

### The present study

The overall goal of this study was to demonstrate the value of adapting a well-validated paper-and-pencil executive function task for VR administration. We developed two VR adaptations of the CTT test: (i) the DOME-CTT, designed for a large-scale VR system, in which the stimuli are projected on a 360° dome-shaped screen surrounding the participant, and (ii) the HMD-CTT, designed for a low-cost head-mount device (HMD), in which the stimuli are presented via VR goggles. In addition to developing the VR-based tests, we evaluated their ability to measure the same cognitive constructs (construct validity) as the gold standard pencil-and-paper CTT, as well as their ability to differentiate among healthy young, middle-aged and older age groups (discriminant validity) relative to the original CTT. Finally, we explored cognitive-motor interactions during performance of the VR-CTT tasks.

## Methods

### General

Two VR-CTT platforms were developed: DOME-CTT and HMD-CTT. Findings from experiments using these platforms are described in Study 1 and Study 2, respectively. There were a total of 147 healthy participants in Study 1 and Study 2 who completed this testing as part of larger experimental protocols (see Additional file 1: Table S1). Participants were subdivided into the following age groups: (1) young adults (YA), ages 18–39 years (n = 50); (2) middle-aged adults (MA) ages 40–64 years (n = 80); and (3) older adults (OLD), ages 65–90 years (n = 17). For all groups, exclusion criteria were motor, balance, psychiatric or cognitive conditions that may interfere with understanding the instructions or completing the required tasks (determined by screening interviews). The protocols were approved by the Sheba Medical Center institutional review board (IRB), and all participants signed informed consent prior to enrolling in the study.

### Methods for Study 1 (DOME-CTT)

#### Participants

Data from 14 YA [age: 27.9 ± 5.0 (mean ± SD) years, education: 16.4 ± 2.9 (mean ± SD) years; 9 females] and 29 MA (age: 55.8 ± 6.2 years, education: 16.3 ± 3.0 years; 16 females) were included in Study 1.

#### Apparatus

**Fig. 1:**
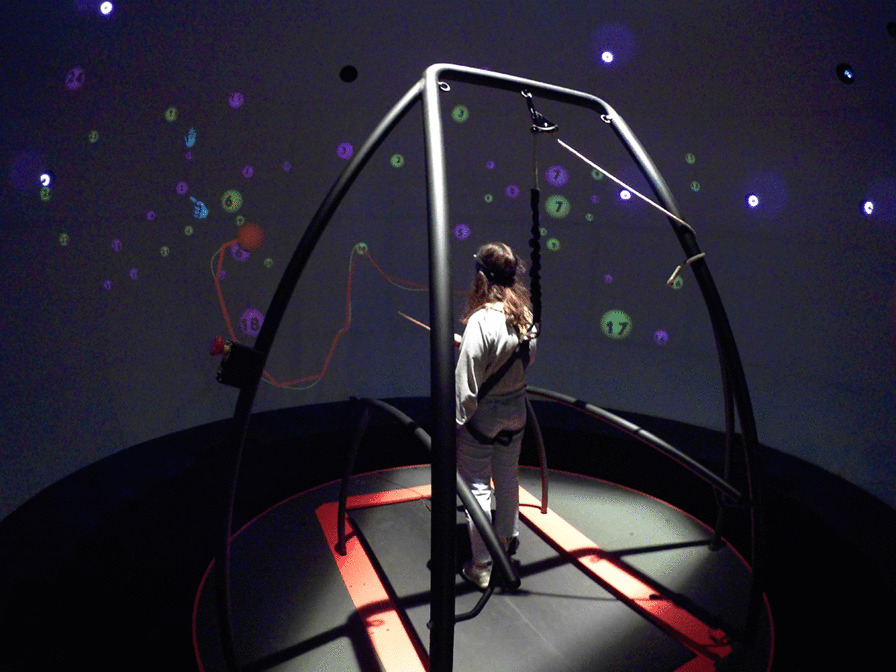
The CAREN High End (Motek Medical, The Netherlands) has a 6-degree of freedom (three translation axes, three rotation axes) moveable platform that is synchronized with a virtual visual scene projected on a 360° dome-shaped screen (projection resolution 1920X1080 lines). A split-belt treadmill is embedded in the platform (not operated in this study). A motion capture system (Vicon) and a pair of force plates embedded in the treadmill provide data on the participant’s spatiotemporal position during the task (sampling rate 120 Hz). The participant who performs the DOME-CTT VR adaptation (see text), is secured with safety harness, holding a wand-like pointing stick with a marker on its edge in order to control the avataric red ball and to move it towards the target ball

A fully immersive virtual reality system (CAREN High End, Motek Medical, The Netherlands) projected a virtual environment consisting of the task stimuli on a full-room dome-shaped screen surrounding the participant (Fig. 1). The system comprises a platform with an embedded treadmill and is synchronized to a motion capture system (Vicon, Oxford, UK). Auditory stimuli and feedback are delivered via a surround sound system.

#### Adapting the pencil-and-paper Color Trails Test for large-scale VR—The DOME-CTT (Fig. 2)

**Fig. 2:**
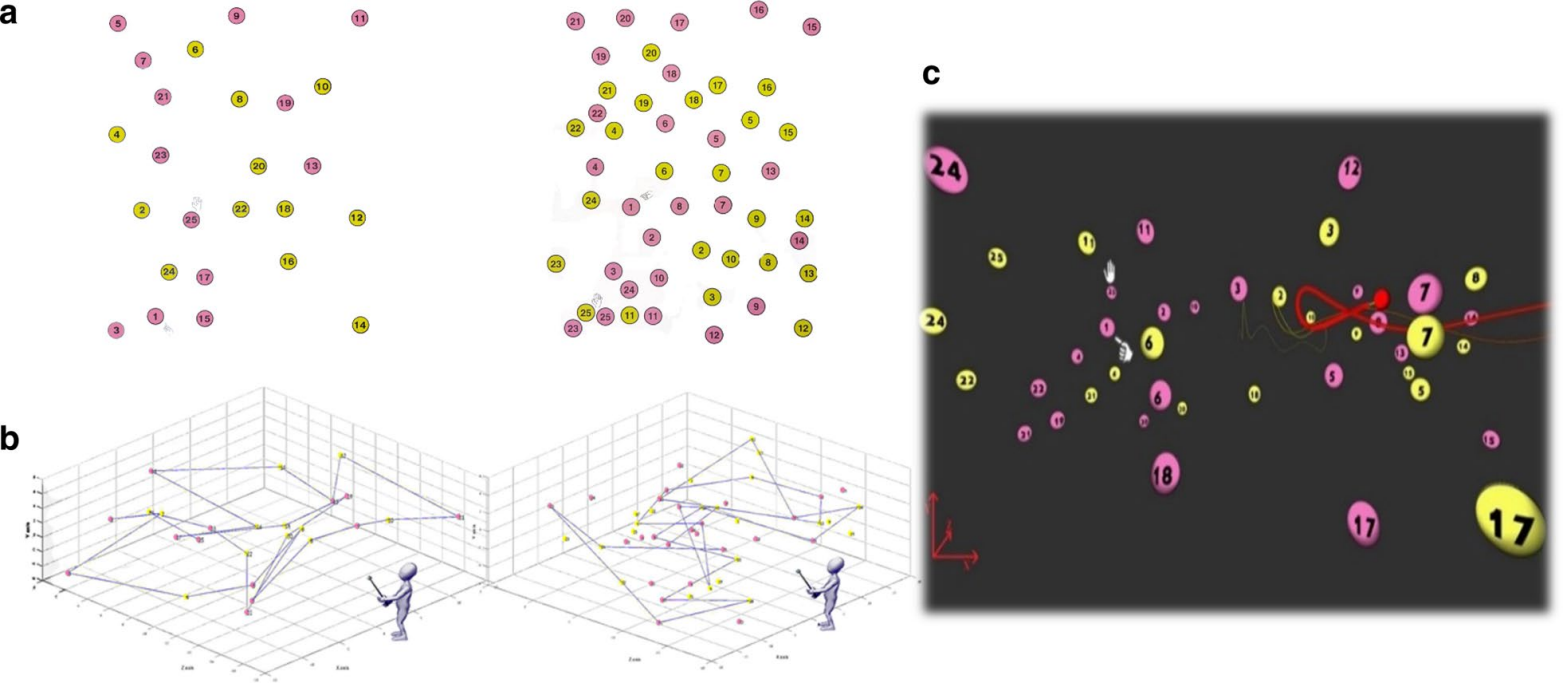
Adaptation of the pencil-and-paper CTT to yield the DOME-CTT.** a** Trails A (left) and B (right) tasks from the pencil-and-paper CTT. The first and last balls are each indicated by a picture of a hand (pointing and stop signing, respectively, seen here in Trails B only). **b** Schematic depiction of the spatial geometric orgnizaiton of the target balls in the three dimensional space. **c** Trails B task from the DOME-CTT, as viewed by the participant. Ball size varies as a function of its proximity to the participant in the VR space. A fixed pointing stick marker is represented by the red ball. Its trace is depicted in yellow, while the red, more pronounced trace is used for visual feedback in the VR space. The first and last balls are indicated using the hand icon

A virtual version of the CTT was developed to demonstrate the feasibility of performing neuropsychological testing in a virtual environment. The original pencil-and-paper CTT consists of four parts: practice (Trails) A, test (Trails) A, practice (Trails) B and test (Trails) B [27]. As below, all were adapted to the VR environment. In the VR version of the CTT, the two-dimensional (2D) page (Fig. 2a) is replaced with a three-dimensional (3D) VR space (Fig. 2b, c) that introduces the dimension of depth to the target balls (that replace the 2D circles) and to the generated trajectory. The translation to 3D geometry followed principles governing the 2D design (compare Fig. 2a and Fig. 2b, c). For example: (1) balls were positioned so that virtual trajectories between sequential target balls would not cross previous trajectories (i.e., between target balls from earlier in the task sequence); (2) proximity of balls in a given region of the 3D space was similar to that in the corresponding region of 2D space in the original CTT; (3) for Trails B, we positioned the corresponding identically-numbered distracter ball of incorrect color at a relative distance to the target ball similar to the that in the original 2D CTT.

The participant performed the DOME-CTT with a marker affixed to the tip of a wand-like pointing stick held in the dominant hand (corresponding to the pen or pencil in the original CTT). The three-dimensional coordinates of the marker were tracked in real time by the motion capture system at a sampling rate of 120 Hz. A virtual representation of this marker appeared within the visual scene (i.e., ‘avatar’, represented by a small red ball—Fig. 2c). To mimic drawing lines in the 2D pencil-and-paper CTT, as the participant moved his/her hand within the VR space, a thick red ‘tail’ trailed directly behind the position of the (red ball) avatar, gradually becoming a faint yellow tail as the avatar moved farther away from the initial position (Fig. 2c).

Movement of the marker was recorded in real time by a motion capture system that allows the reconstruction of kinematic data over the duration of the test.

The testing procedure was also adapted for the new format. As above, the original pencil-and-paper CTT comprises four consecutively administered test levels: (1) Trails A practice; (2) Trails A; (3) Trails B practice; and (4) Trails B [16]. Though drawing lines with a pen/pencil on a piece of paper is highly familiar, manipulation of the VR ‘controller’ (i.e., the marker affixed to the pointing stick) to move an avatar (i.e., the red ball) within the virtual environment is a relatively unfamiliar skill. Thus, the DOME-CTT began with an additional practice level in which participants practiced guided movement of the avatar within the virtual space to so that it touched the numbered ball targets. During this level, participants were introduced to the positive feedback received when the avatar ball touched the correct ball (i.e., momentary enlargement of the ball) and the negative feedback when it touched an incorrect ball (i.e., brief buzzing sound). These feedback stimuli were also presented during the remainder of the testing session. After this initial practice level, test levels corresponding to those in the original CTT were administered. However, unlike the pencil-and-paper CTT, Trails A and Trails B were each preceded by two different practice levels. In the first practice level, all virtual balls were clustered near the center of the visual field, and in the second practice level, the balls were distributed throughout the visual field, approximating the spatial distribution of the balls in the actual testing levels. A video demonstration of the DOME-CTT is provided in Additional file 2.

#### Procedure

Data on pencil-and-paper CTT and DOME-CTT were collected as part of three different experimental protocols (see Additional file 1: Table S1). All data (with the exception of test retest data) described in this study were collected on the first visit. The participants completed the pencil-and-paper CTT and DOME-CTT on the same day in counterbalanced order across participants. We monitored the general wellbeing of the participants (e.g., absence of fatigue) throughout the tests.

#### Outcome measures and statistical analysis

For the pencil-and-paper CTT and the DOME-CTT, completion times for Trails A and B were recorded (t_A_, t_B,_ respectively). Construct validity was assessed by correlating t_A_ and t_B_ from the DOME-CTT with the corresponding scores from the gold standard CTT (Pearson coefficient). Analysis of variance (ANOVA) was used to assess effects of Group (young, middle aged; between-subjects factor), Trails (Trails A, Trails B; within -subjects factor) and Format (pencil-and-paper CTT, DOME-CTT; within-subjects factor). Partial Eta Squared was computed as a measure of effect size. To verify suitability of parametric statistics, Shapiro–Wilk normality tests were run for each outcome variable per group. Of the eight normality tests, none indicated non-normal distributions (Shapiro–Wilk statistic ≤ 0.93; *p* ≥ 0.16). Levene's test [29] revealed inhomogeneity of variance among groups for Trails A and B in pencil-and-paper and VR formats (*p* < 0.05). Therefore, the data were log-transformed prior to applying ANOVA tests. On the new data sets we confirmed homogeneity of variance assumption for Trails A and B of the pencil-and-paper CTT and for Trails B of the DOME-CTT (*p* > 0.05). Descriptive statistics, figures and correlations analyses were performed on the pre transformed data.

Summary statistics (mean ± SD) were computed for t_A_ and t_B_ from the pencil-and-paper CTT and DOME-CTT.

Errors were manually recorded by the experimenter for the pencil-and-paper CTT [27]; for the DOME-CTT, errors were recorded both manually and automatically by the software. Related samples Wilcoxon Sign Test (non-parametric) test was used to evaluate the Format effect separately for Trails A and B. Mann–Whitney *U* tests were used to evaluate the group effect.

To examine discriminant validity (i.e., ability to separate between YA and MA) of the DOME-CTT as compared with the pencil-and-paper CTT, we plotted receiver operating characteristic curves (ROC) for Trails A and Trails B (i.e., t_A_ and t_B,_ respectively) for each test format and calculated the area under the curve (AUC; range: 0–1, higher values reflect better discriminability).

Level of statistical significance was set at 0.05. Statistical analyses were run using SPSS software (SPSS Ver. 24, IBM).

### Methods for Study 2

#### Participants

Data from 36 YA (age: 26.7 ± 4.1 [mean ± SD] years, education: 15.9 ± 2.3 [mean ± SD]; 21 females), 51 MA (age: 56.2 ± 6.2 years, education: 16.8 ± 3.0 years; 39 females) and 17 OLD (age: 73.7 ± 6.5 years, education: 13.1 ± 2.7 years*;* 11 females) were included in Study 2.

#### Apparatus

VR technologies have advanced rapidly in recent years. In addition to new technical features for precise stimulus delivery and response measurement, as well as enhanced usability, low-cost VR is now widely accessible. The most accessible type of VR system is the head-mount device (e.g., HTC Vive, Oculus Rift), which is designed for home-based operation. In addition to its continued popularity for entertainment, VR is now being applied in a variety of ‘serious’ contexts, ranging from surgical simulation to the study of human performance and psychological function [19, 30]. For this study, we used a fully immersive VR system (HTC-Vive; New Taipei City, Taiwan) including a headset with ~ 100° field of view (FOV) in the horizontal plane and ~ 110° FOV in the vertical plan. Also included were a controller for user interaction with the virtual environment and two *‘*lighthouse*’* motion trackers for synchronizing between actual controller position and corresponding position in the virtual environment.

#### Adapting the pencil-and-paper Color Trails Test for a headset-based VR system—The HMD-CTT

**Fig. 3:**
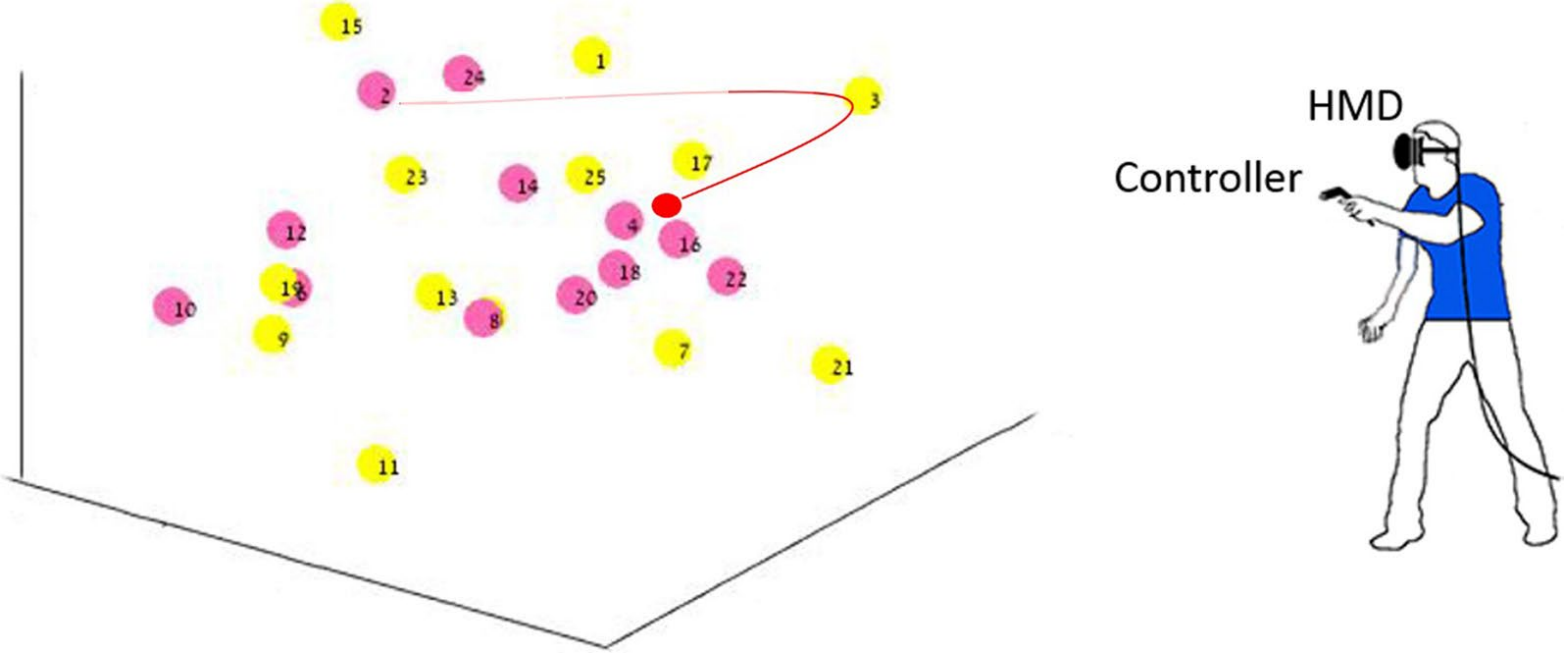
Translation of pencil-and-paper CTT to HMD-based VR format. In the three-dimensional VR-based HMD-CTT (HTC-Vive, projection resolution 2160X1200 lines), the participant completes the task while standing and wearing a VR headset. S/he holds a VR controller in his/her dominant hand to move the (red ball) avatar to sequentially connect numbered balls distributed throughout the virtual space visible in the headset. (Trails A shown.)

In developing the HMD-CTT version, we adopted a similar approach to the development of the DOME-CTT. Briefly, we used the popular Unity3D VR game engine [31] to design a virtual environment for the CTT. With the exception that the participant held the HTC controller rather than a wand-like pointing stick, task design matched the DOME-CTT, including positive and negative feedback cues, practice and test procedures. Figure 3 illustrates how the 2D format of the original CTT was translated to the 3D HMD-CTT format (Trails A; see also video demonstration in Additional file 3). The HMD-CTT incorporated practice and test levels corresponding to those in the DOME-CTT described above.

#### Procedure

The procedure was identical to that of Study 1 (see above).

#### Outcome measures and statistical analyses

For the pencil-and-paper CTT as well as the HMD-CTT, completion times for Trails A and B were recorded (t_A_, t_B,_ respectively). Similar to Study 1, construct validity of t_A_ and t_B_ was assessed by correlating t_A_ and t_B_ from the HMD-CTT with the corresponding scores from the gold standard pencil-and-paper CTT. As in the DOME-CTT study, repeated-measures ANOVA was used to assess the effects of Group (YA, MA, OLD; between-subjects factor), Trails (Trails A, Trails B; within-subjects factor) and Format (pencil-and-paper CTT, HMD-CTT; within-subjects factor). Partial Eta Squared was computed as a measure of effect size. We used the Bonferroni correction to adjust for multiple comparisons in the post-hoc pairwise comparisons.

As for Study 1, Shapiro–Wilk normality tests were run for each outcome variable per group to verify the suitability of parametric statistics. Of the twelve normality tests, four indicated non-normal distributions: t_A_ pencil-and-paper CTT, MA group, Shapiro–Wilk statistic = 0.786, *p* = 0.002; t_B_ pencil-and-paper CTT, MA group, Shapiro–Wilk statistic = 0.770, *p* = 0.002; t_B_ pencil-and-paper CTT, YA group, Shapiro–Wilk statistic = 0.744, *p* = 0.001, t_A_ pencil-and-paper CTT, OLD group, Shapiro–Wilk statistic = 0.844, *p* = 0.015. As in Study 1, Levene's test [29]. revealed inhomogeneity of variance among groups for Trails A and B in pencil-and-paper and VR formats (*p* < 0.05). Thus, the data were log-transformed prior to applying ANOVA analyses, and homogeneity of variance was confirmed for paper-and-pencil Trails A and B (*p* > 0.05).Descriptive statistics, figures and correlations analyses were performed on the pre transformed data. Additional analyses are described in the *Results* section.

Regarding prevalence of errors, related samples Wilcoxon Sign Test (non-parametric) test was used to evaluate the Format effect separately for Trails A and B. Kruskal–Wallis tests were used to evaluate the group effect (three levels) separately for Trails and Format.

As for Study 1, level of statistical significance was 0.05, and statistic analyses were run with SPSS.

#### Qualitative analysis of manual performance

Spatial coordinates of the controller position (corresponding to ‘red ball’ avatar) were recorded throughout the HMD-CTT sessions. Custom software written in MATLAB (Mathworks, Inc.) used this data to extract and analyze the 24 target-to-target reaching movements during Trails A and Trails B, respectively (errors were excluded from this analysis). We made a qualitative assessment of the trajectories generated in each of the three groups by examining the grand averages of their velocity profiles to characterize upper-limb motor behavior associated with the HMD-CTT tasks. For a full description of the methodology used to generate these grand averages, see Additional file 1*.*

### Evaluation of test–retest reliability (Study 1 and Study 2)

To evaluate test–retest reliability, some participants completed a second assessment.

Fifteen MA participants from Study 1 completed an additional evaluation about 12 weeks after the initial evaluation (per protocol 1 in Additional file 1: Table S1) during which they completed the pencil-and-paper CTT and DOME-CTT in the same order as in the initial evaluation.

Thirty-two MA participants from Study 2 completed an additional evaluation about 12 weeks after the initial evaluation. Also from Study 2, twenty participants (*n* = 10 YA, *n* = 1 MA, *n* = 9 OLD) completed an additional evaluation 2 weeks after the initial one. The pencil-and-paper CTT and HMD-CTT were administered in the same order as in the initial evaluation.

To assess test–retest reliability, we computed intraclass correlation coefficients (ICC; two-way mixed, effects, absolute agreement, [32]) for t_A_ and t_B_ scores from the traditional pencil-and-paper CTT and the DOME-CTT (*Study 1*) or HMD-CTT (Study 2) collected at two visits. ICC reflects similarity of the obtained scores irrespective of the level of performance, reflecting not only correlation, but also agreement between measurements [33, 34]. By convention ICC > 0.75 is considered good reliability [32].

## Results

### Study 1

#### Performance on the DOME-CTT: group, trails and format effects

All participants completed the tests in both formats. Time for initial DOME-CTT practice levels varied between participants, but usually did not exceeded 10–15 min. Due to technical malfunction, data for DOME-CTT Trails A from one YA participant was not recorded.

Statistical analysis revealed effects of Group (F_1,40_ = 25.4, *p* < 0.001, η^2^ = 0.38; longer completion time for middle-aged), large effects of Trails (F_1,40_ = 273.7, *p* < 0.001, η^2^ = 0.87; longer completion time for Trails B) and large effects of Format (t_A_: F_1,40_ = 1301.7, *p* < 0.001, η^2^ = 0.97; longer completion time for DOME-CTT). The Format X Trails interaction was also found to be significant (F_1,40_ = 19.5, *p* < 0.001, η^2^ = 0.32,), reflecting a larger difference between Trails A and B for the DOME-CTT (i.e., as compared to the pencil-and-paper CTT. None of the other interactions (i.e., Group X Format, Group X Trails X Format and Group X Trails) were statistically significant (*p* ≥ 0.09).

**Fig. 4:**
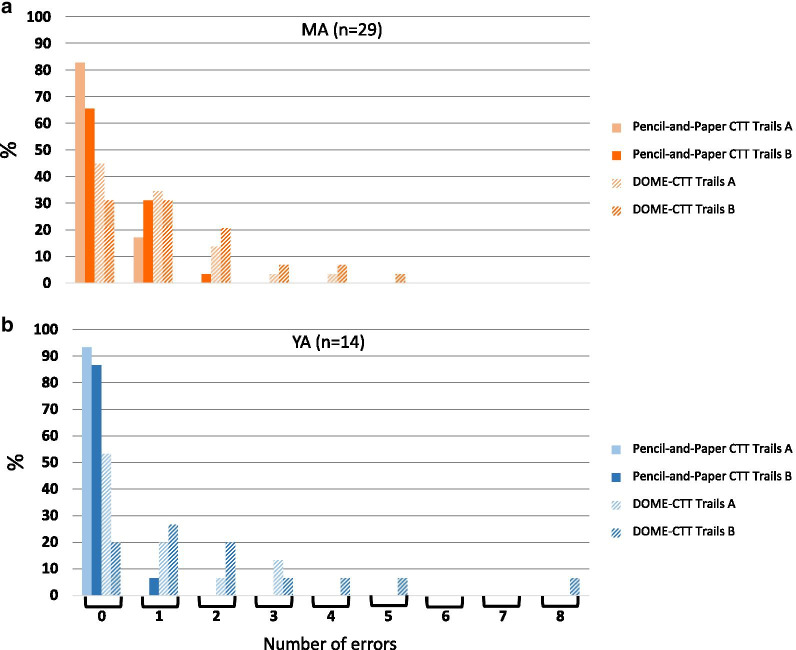
Comparison of error rates between pencil-and-paper CTT and DOME-CTT VR-based adaptation. Percent of participants making 1, 2, 3, 4, 5, 6, 7, or 8 errors for paper-and-pencil and DOME-CTT versions of Trails A and Trails B tasks (see key), separately for the MA group (**a**) and the YA group (**b**). For the pencil-and-paper CTT, most participants in both groups made no errors, and none made more than 2 errors. Conversely, for the DOME-CTT, less than half of the participants (YA and MA combined) made no errors and some made a substantial number of errors

Errors were more prevalent during performance of the DOME-CTT (Fig. 4) for both Trails A and B (*p* < 0.001). The group effect did not reach statistical significance (*p* ≥ 0.056). The MA group made more errors than the YA group irrespective of Trails and Format (*p* ≤ 0.002).

#### Correlations between pencil-and-paper and DOME-CTT completion times

**Fig. 5:**
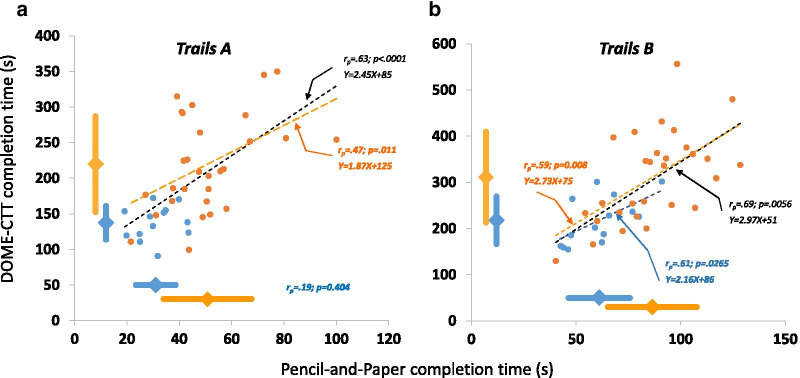
Convergent construct validity of the VR-based DOME-CTT. Trails A (*t*_*A*_, left panel) and Trails B (*t*_*B*_, right panel) completion time recorded during the gold-standard pencil-and-paper CTT plotted against the corresponding completion times recorded during the DOME-CTT for YA (blue) and MA (orange) participants. One YA datapoint is missing for Trails A, as the participant did not complete the DOME-CTT (i.e., due to technical malfunciton). Pearson correlations are shown for YA, MA, as well as combined (black) groups, and regression lines are plotted for significant correlations. Diamond markers and thick lines adjacent to the axes indicate mean ± SD for YA and MA groups

Figure 5 shows the relationship between performance on the pencil-and-paper and DOME-CTT for the YA (blue) and MA (orange) groups. The Spearman correlation (rho; *r*_*s*_) between Part A completion time (t_A_) on the gold-standard pencil-and-paper CTT and the corresponding Part A completion time on the DOME-CTT was 0.58 (*p* < 0.001; Fig. 5a). For Part B completion time (t_B_), the Spearman correlation was 0.70 (*p* < 0.001; Fig. 5b).

### Study 2

#### Performance on the HMD-CTT: group, trails and format effects

One OLD participant (age: 70 years, education: 12 years, female) could not perform the HMD-CTT practice and actual test levels, showing general disorientation. Another participant from this group (age: 89 years, education: 12 years, male) asked to stop the HMD-CTT during the Trails B portion, expressing frustration at his perceived poor performance (see also Fig. 7 legend). Data from these participants were omitted from relevant analyses.

Statistical analysis revealed significant effects of Group (F_2,98_ = 41.9, *p* < 0.001, η^2^ = 0.46; progressively longer completion time with more advanced age, all pairwise comparisons p < 0.001), Trails (F_1,98_ = 617.2, *p* < 0.001, η^2^ = 0.86; longer completion time for Trails B) and Format (F_1,98_ = 660.8, p < 0.001, η^2^ = 0.87; longer completion time for HMD-CTT). Of the interaction effects, only the Format X Trails interaction was significant (F_2,98_ = 14.3, *p* < 0.001, η^2^ = 0.12) due to a larger Trails effect for the HMD-CTT vs. the pencil-and-paper CTT. The Group x Format, Group X Trails and Group X Format X Trails interactions were not significant (p > 0.30).

As participants in the OLD group had significantly fewer years of education than participants in the YA and MA groups (p ≤ 0.001; see *Methods*), we repeated the analysis entering years of education as a covariate. The results did not change appreciably (see Additional file 1).

**Fig. 6:**
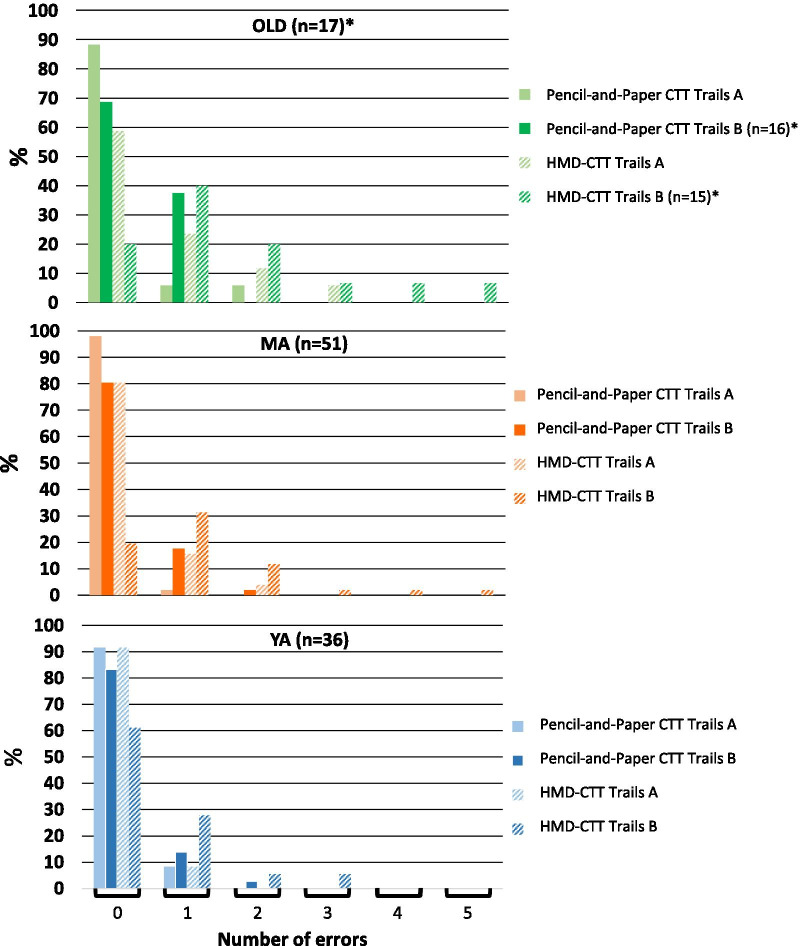
Comparison of error rates between pencil-and-paper CTT and HMD- CTT VR-based adaptation. Percent of participnats making 1, 2, 3, 4, or 5 errors for paper-and-pencil and HMD-CTT versions of Trails A and Trails B tasks (see key), separately for OLD (top panel), MA (middle panel), and YA (bottom panel) groups. Similar to the DOME-CTT (Fig. 5), substantially more errors were made for the VR-based HMD-CTT than for the paper-and-pencil CTT; this tendency was particularly evident for OLD participants

Errors were more prevalent during performance of the HMD-CTT (Fig. 6, *p* ≤ 0.004). Interestingly, a significant group effect was found only for HMD-CTT (Trails A and B; H ≥ 8.8, *p* ≤ 0.012) but not for the pencil-and-paper CTT (H ≤ 2.96; *p* ≥ 0.227). Post-hoc analyses revealed that the significant Group effect for HMD-CTT was attributable to more errors among the OLD than the YA (*p* = 0.009).

#### Correlations between pencil-and-paper and HMD-CTT—completion times

**Fig. 7:**
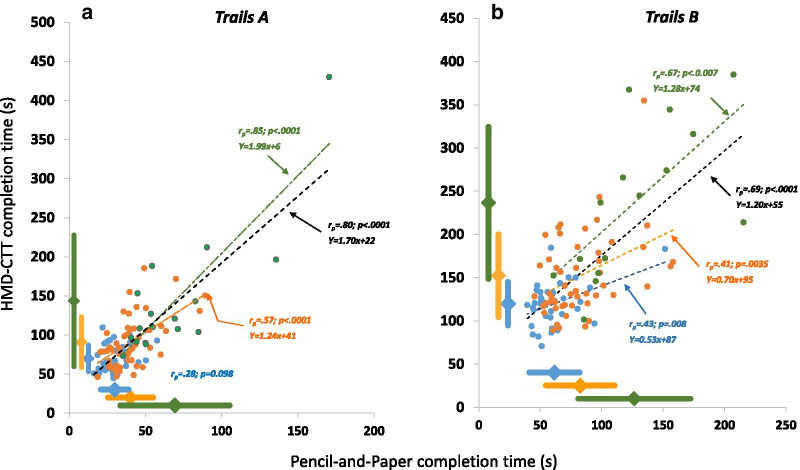
Convergent construct validity of the VR-based HMD-CTT. Trails A (*t*_*A*_, left panel) and Trails B (*t*_*B*_, right panel) completion time recorded during the gold-standard pencil-and-paper CTT plotted against the corresponding completion times recorded during HMD-CTT, for YA (blue), MA (orange), and OLD (green) participants. Two OLD datapoints are missing as the participants did not complete the HMD-CTT.*Pearson correlations are shown for YA, MA, OLD and combined (black) groups, and regression lines are plotted for significant correlations. Dimond markers and thick lines adjacent to the axes indicate mean ± SD for YA, MA, and OLD groups. *One OLD participant who could not finish the HMD-CTT Trails B had the slowest Trails A completion time, for both pencil-and-paper (i.e., 170.5 s) and HMD versions (450.2 s). His completion time for the pencil-and-paper Trails B was 327.3 s (this data point was not plotted)

Figure 7 shows the relationship between performance on the pencil-and-paper and HMD-CTT for the YA (blue), MA (orange) and OLD (green) group. The Spearman correlation (rho; *r*_*s*_) between Part A completion time (t_A_) on the gold-standard pencil-and-paper CTT and the corresponding Part A completion time on the HMD-CTT was 0.62 (*p* < 0.001; Fig. 7a). For Part B completion time (t_B_), the Spearman correlation was 0.69 (*p* < 0.001; Fig. 7b).

#### Qualitative analysis of manual performance

**Fig. 8:**
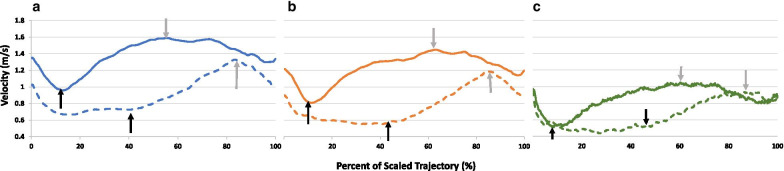
Grand averages of HMD-CTT hand movement velocity profiles. HMD-CTT hand movement velocity (meters/second) profiles showing trajectories for young (**a**), middle-aged (**b**), and older adult (**c**) groups for Trails A (solid line) and Trails B (dashed lines) over time, scaled to reflect percent from trajectory completion duration. In **a** (YA), for each of Trails A and B, 840 trajectories were avaraged from 35 participants; in **b** (MA), 1176 trajectories were averaged from 49 participans; in **c** (OLD), 336 trajectories were averaged from 14 participants. See *text* for additional description and interpretation

Figure 8 shows grand averages of the scaled ball-to-ball hand trajectory velocity profiles (see methodologies in Additional file 1) for all participants who completed HMD-CTT Trails A (solid traces) and B (dashed traces). Data for the three age groups is color-coded (see legend for details). Based on these traces, the following observations can be made:

1. For all groups and for both Trails A and B, movement toward a target (ball) does not stop immediately upon reaching the target (virtually touching, at x = 100% which for all, except for the last trajectory, is also x = 0% of the next trajectory), but little later, as reflected by the ensuing gradual decrease in velocity decrease at x = 0%, reaching a minimum and ensuing gradual increase in velocity. This initial decrease on the grand average traces does not reach zero because the minimum is reached at a different time for each of the 24 individual ball-to-ball trajectories (see Additional file 1: Fig. S1).
2. For Trails A, but not for Trails B, soon after reaching this minimum (i.e., completing the execution of the previous trajectory), a new trajectory can be identified (time of emergence is designated by the left black arrow). The velocity profile of this trajectory is characterized by an accelerating portion (peak indicated by a gray arrow) and a decelerating portion upon approaching the target ball. The degree of asymmetry between these portions of the trajectory varies between groups, with YA showing greater symmetry.
3. Conversely, for Trails B, a prolonged ‘executive function’ period is evident, and acceleration toward the target is identifiable after at least 40% of the trace, with the older groups showing a more delayed initiation of movement (black arrows on dashed traces). This pattern is consistent with the divided attention aspect of Trails B, in which the participant must locate the ball next in the numerical sequence but of opposite color to the current ball, ignoring the distracter ball with the correct number but the incorrect color (i.e., same color as the current ball).
4. Consistent with the results for completion times (t_A_, t_B_; Table 2), the velocity profiles for Trails A are faster than those of Trails B.

### Test–retest reliability (Studies 1 and 2)

For the DOME-CTT (retest interval of about 12 weeks), moderate reliability was found: Trails A (t_A_) and B (t_B_) (ICC = 0.597, *p* = 0.023, ICC = 0.676, *p* = 0.018, respectively). For the pencil-and-paper CTT, comparatively better reliability was found for Trails A (t_A_) (ICC = 0.778, *p* = 0.001) and Trails B (t_B_) (ICC = 0.622, *p* = 0.040).

For the HMD-CTT (retest interval of about 12 weeks), moderate reliability was found for Trails A (t_A_) and B (t_B_) (ICC = 0.618, *p* = 0.004; ICC = 0.593, *p* = 0.003, respectively). For the pencil-and-paper CTT, comparatively better reliability was found for Trails A (t_A_) and Trails B (t_B_) (ICC = 0.744, *p* < 0.001; ICC = 0.769, *p* < 0.001, respectively).

In both VR-CTT formats, there were no substantial differences in ICC values for MA participants who engaged in cognitive training during the 12 week period (as part of the larger study protocol; see Additional file 1: Table S1) as compared with those who did not (see details in section D of Additional file 1).

For the HMD-CTT (retest interval of about 2 weeks), good reliability was found for Trails A (t_A_) and B (t_B_) (ICC = 0.748, *p* = 0.002; ICC = 0.893, p < 0.001, respectively). The paper-and-pencil CTT also showed good reliability for both Trails A (t_A_) and Trails B (t_B_); Compared to HMD-CTT, the gold standard test had higher reliability for t_A_ and lower reliability for t_B_ (ICC = 0.851, p < 0.001; ICC = 0.798, *p* = 0.001, respectively).

### Discriminant validity (Studies 1 and 2)

Table 3 shows AUC values from ROC curves (shown in Additional file 1). Pencil-and-paper CTT AUC values were compared with AUC values obtained for each of the VR adaptations (i.e., DOME-CTT, HMD-CTT). For each VR adaptation, the relative difference [%] in AUC from the pencil-and-paper AUC is shown. Comparisons were made separately for Trails A and Trails B.

The data indicate that all CTT versions have relatively high discriminant validity (AUC ≥ 0.70; *p* ≤ 0.05). AUCs are largely comparable, though slightly reduced for the VR adaptations.

#### Comparing completion times between DOME-CTT and HMD-CTT

The completion times in Tables 1 and 2 suggest that t_A_ and t_B_ are higher (i.e., longer) for DOME-CTT relative to HMD-CTT. None of the participants completed both DOME-CTT and HMD-CTT testing, and the present work was not designed to compare between the two VR platforms. However, we conducted analyses to address this question based on the existing evidence. The methodology and the results detailed in the supplemental material (Additional file 1) suggest shorter completion times for HMD-CTT as compared to the DOME-CTT (Tables 1 and 2).

**Table 1:** Mean values for CTT completion times (mean ± SD)

	Pencil-and-Paper CTT	DOME-CTT
YA: t_A_ (s)	31.01 ± 7.62	137.39 ± 23.84
YA: t_B_ (s)	61.09 ± 14.66	213.16 ± 53.18
MA: t_A_ (s)	50.81 ± 16.80	219.87 ± 67.31
MA: t_B_ (s)	86.53 ± 21.22	311.26 ± 98.61

*YA* Young adults; *MA* Middle Aged, *s* seconds; t_A_ completion time of Trails A; t_B_ completion time of Trails B

**Table 2:** Mean values for CTT completion times (mean ± SD)

	Pencil-and-Paper CTT	HMD-CTT
YA: t_A_ (s)	29.78 ± 9.08	69.93 ± 17.03
YA: t_B_ (s)	61.52 ± 20.53	119.82 ± 25.23
MA: t_A_ (s)	40.15 ± 15.02	91.36 ± 32.60
MA: t_B_ (s)	82.60 ± 27.88	152.24 ± 47.99
OLD: t_A_ (s)	63.55 ± 35.95	126.75 ± 84.26
OLD: t_B_ (s)	126.57 ± 45.92	236.52 ± 88.00

*YA* Young adults, *MA* Middle Aged, *EA* Elderly adults, *s* seconds; t_A_ completion time of Trails A; t_B_ completion time of Trails B

## Discussion

In this report, we describe the development and initial validation of VR-based adaptations of the Color Trails Test (CTT) [27], a traditional pencil-and-paper test of attention and processing speed, using two different types of VR systems. The first VR-based system involves projection of visual stimuli on the walls of a large, dome-shaped room (akin to a cave, monoscopic projection). The second VR-based system is a low-cost head-mount VR device (HMD) worn by the participant and suitable for home-based assessment. Adherence and usability of both VR-based adaptations proved to be relatively good, with only two participants (~ 1.5%) not completing the VR tasks. Participants only rarely complained about the difficulty of completing the VR tasks, though there were no such complaints for the pencil-and-paper version. Our discussion integrates the results from two studies, each of which evaluated one of the VR-based adaptations.

### Construct validity

Our results suggest that the new VR-based adaptations and gold standard pencil-and-paper version share similar psychometric properties (e.g., longer completion time for B vs. A). Coupled with the relatively high correlations between corresponding parts (~ 0.7; Figs. 5 and 7), this suggests that the VR and the pencil-and-paper tests measure the same cognitive constructs (e.g., sustained, divided attention). By comparison, in a cross-validation study of the TMT and CTT, Dugbartey and colleagues reported lower correlation values of 0.35 for Trails A and 0.45 for Trails B [35].

Notably, construct validity correlations for YA participants on the Trails A portion of the test were not significant. We attribute this result to a ceiling effect in that t_A_ values approached the shortest completion times technically possible on all three CTT tests.

### Completion time: format effects

Trails A and Trails B completion times were significantly longer for the VR-based adaptations compared with the pencil-and-paper CTT, possibly reflecting a larger dynamic range of performance for the VR versions (e.g., even if only attributable to the larger spatial area covered by the VR task as compared to one page distribution of the targets in the pencil-and-paper CTT), greater task difficulty [36] and/or the cognitive-motor interactions relevant to the VR versions but not the original pencil-and-paper test.

Perceptual factors must also be considered. While the participant has an egocentric viewpoint for both pencil-and-paper and VR-based versions [37], s/he likely has different perceptions of the candidate actions available to perform the task (i.e., different perception of affordances) [38–40]. Presumably, during the pencil-and-paper test, visual scanning is mainly by saccadic eye movements and short-distance visual pursuits. In contrast, the VR versions require head “gaze” (i.e., motor programs for the neck and upper trunk muscles to execute head rotations mainly around the yaw and pitch axes) combined with longer ocular pursuits and with saccades for operational visual scanning. Further, in the pencil-and-paper CTT, motor activity of the hand is limited to drawing short lines between the printed circles. However, in the VR-CTT versions, larger arm reaching movements are required as well as postural adjustments and occasional stepping (multi directional). It is conceivable that prior to a given task and during the short practice levels, the participant ‘tunes’ his/her perception of the affordance related to the task, which includes the more complex integration required for the various actions associated with the VR-CTT versions.

Finally, we speculate that the three-dimensional target layout within a black space with perceived infinite boundaries, i.e., unknown physical limits of the VR-CTT versions as compared with the finite boundaries of the pencil-and-paper CTT version, contributes to longer test execution times. Specifically, the participant may have difficulty with movement scaling in the absence of physical reference boundaries on the VR-based versions. This speculation can be tested in future studies by the use of a VR version that includes virtual physical boundaries (e.g., target balls floating in a room).

Among the two VR adaptations, completion times for the DOME-CTT were longer than those for the HMD-CTT (compare Tables 1 and 2, Figs. 5 and 7; post-hoc analyses comparing across studies—Additional file 1). One possible account for this finding relates to different levels of visual immersion between the tests. The HMD-CTT provides no visual feedback from the arms, and the participant’s subjective experience consists solely of moving the avatar (red ball) within the VR environment. In contrast, during the DOME-CTT, the participant sees his/her hand holding the wand-like stick in addition to the virtual avatar as s/he makes reaching movements toward the target balls. The latter configuration may complicate sensorimotor integration given the two parallel, relevant sensory input streams (physical hand, virtual avatar). A potential contributor to this complication is that participants can see their arms in full stereoscopic vision but the balls only in monoscopic projection.

Subramanian and Levin reported superior motor performance (reaching movements) among healthy adults in a large-scale screen-based VR system as compared to an HMD-based system [41], apparently at odds with the present findings (i.e., slower movements for DOME-CTT as compared with HMD-CTT). Likely technical-methodological differences between the studies account for the disparity. For example, the HMD field of view was smaller (~ 50° vs. ~ 100°) in the Subramanian and Levin study, and the type of task (reaching vs. consecutive trails making following rules) was markedly different.

### Error performance

Participants made significantly more errors (i.e., touching the wrong ball) on the VR-based versions as compared to the pencil-and-paper CTT (Figs. 4 and 6). For example, only about 14% of all pencil-and-paper test levels completed in Study 2 (Trials A and B across all three cohorts) had at least one error (most often one error). The error rate was 2.5 times higher for HMD-CTT test levels (~ 35%).

This pattern of results may seem paradoxical, as with longer completion times on the VR-based tests, fewer errors should occur, but our data reflect the opposite. Indeed we believe that as the VR tasks are more demanding then the corresponding pencil-and-paper tasks, the cognitive processes classically associated with the CTT paradigm might be compromised, leading to more errors [42–45]. Specifically, we posit that the VR-based tasks make much greater demands on motor planning and execution (see below), visual scanning and spatial orientation, and involve higher perceptual and/or cognitive load. Apparently, this load differentially affected elderly as compared to YA, as evidenced by the significantly higher error rates for the OLD group.

### Cognitive-motor interactions

Our qualitative analyses clearly demonstrate that when shifting from a cognitive task primarily involving sustained attention (Trails A) to one that primarily involving divided attention (Trails B), upper-limb motor behavior changes (Fig. 8). Previous research has employed VR to evaluate cognitive-motor interactions mainly in the context of locomotion. Most of the studies have reported clinical benefits related to cognitive-motor interactions associated with immersion in a VR environment [46–50].

In the current study, we began exploring the effect of divided attention (operationalized as HMD-CTT Trails B performance) on the planning and execution of upper-limb reaching movements. The well-documented single-peak velocity profile typical of ballistic movements [51–53] appears to govern the hand trajectories generated during HMD-CTT Trails A, a sustained attention task, but not during Trails B. In Trails B, a divided attention task, trajectories are characterized by an initial slow increase in the velocity profile, probably reflecting neural processes more related to executive function and less to motor execution. Potential age effects (e.g., less symmetric peak, slower overall velocity) are apparent in comparing the velocity profile across age groups (Fig. 8).

Follow up studies will focus on developing reliable quantification methods and metrics to assess these cognitive-motor interaction effects. Notably, comparing the velocity profiles generated during a three-dimensional (3D) VR-based task to a classical two-dimensional (2D) task as the ‘gold standard’ [54] is suboptimal, mainly due to the absence of a reliable theoretical model for three-dimensional hand-reaching movements. Thus, new referencing methodologies like sampling single target-to-target trajectories should be included as part of future versions and analyses of VR-based CTT tasks like those used here.

### Discriminant validity

The VR-based tests were largely comparable though not superior to the pencil-and-paper CTT in terms of distinguishing individuals of different age-groups on the basis of CTT completion time (t_A_ and t_B_, Table 3). Further, both the traditional and VR-based versions demonstrated relatively high discriminant validity as reflected by high AUC values. These observations are consistent with the strong correlations for completion times between the VR-based and original CTT for each age group (with the exception of t_A_ in YA) as well as when combining participants across age groups (black dashed lines in Figs. 5 and 7).

**Table 3:** AUC values from ROC curves

	Pencil-and-Paper-CTT		DOME-CTT	
	Trails A (t_A_)	Trails B (t_B_)	Trails A (t_A_)	Trails B (t_B_)
YA vs. MA	0.88**	0.82**	0.88** [0%]	0.81** [− 2%]

	Pencil-and-Paper-CTT		HMD-CTT	
YA vs. MA	0.73*	0.77*	0.70* [− 5%]	0.71* [− 8%]
YA vs. OLD	0.95*	0.95*	0.92* [-4%]	0.92* [− 4%]
MA vs. OLD	0.83**	0.80**	0.75** [-10%]	0.80** [0%]

*p = .05; ** p < .0003; []—relative difference of AUC for VR adaptation as compared with Pencil-and Paper CTT

*AUC* area under the curve, *ROC* receiver operating characteristic curves, *YA* young adults, *MA* middle aged adults, *OLD* Elderly adults

Comparability in discriminant validity between VR-based and the gold standard CTT for completion times is encouraging. However, VR-based testing affords additional metrics that may be better at differentiating among age groups as well as between healthy and cognitively impaired individuals. Indeed, VR facilitates the development of new parameters of greater relevance to daily living (i.e., more ecologically valid) in that they better capture complex, integrated behaviors. Thus, we speculate that using such VR-based parameterization of multimodal function (e.g., hand-gaze coordination combined with hand trajectories) will provide superior discriminant validity.

### Test retest reliability

For a retest period of ~ 12 weeks, the VR-based CTT adaptations showed moderate reliability (intraclass correlation of ~ 0.6), while the pencil-and-paper version showed generally better reliability. The superior reliability of the original CTT for this retest interval may be attributable to the greater familiarity of the pencil-and-paper format, which may have led to a larger learning effect upon retest and consequently poorer reliability for the VR-based versions (see [55]). We also acknowledge that some middle-aged participants had engaged in a cognitive training protocol during the 12-week interval (see Additional file 1: Table S1) which may compromise test- retest evaluations.

However, for a retest period of ~ 2 weeks, both the HMD-CTT and the original CTT showed good reliability (intraclass correlation of ≥ 0.75), with the VR-based adaptation showing superior reliability for Trails B. Our results are consistent with findings that shorter retest intervals yield higher reliability coefficients [56]. As there does not appear to be a clear convention for the ideal test–retest interval [57], our data reporting reasonable reliability for both intervals is relevant and informative. Still, given that our sample sizes were small, the findings should be replicated in larger studies.

### Limitations

This study had several notable methodological/technical limitations. Some concepts could not be directly translated from the pencil-and-paper CTT to the VR-CTT versions. For example, we provided positive feedback upon reaching the correct target ball in the VR versions, while only negative feedback is provided pencil-and paper version (i.e., when drawing a line to the wrong circle). Our decision to provide positive feedback in the VR versions was to assure the participant that the target had ben successfully reached. In addition, to familiarize the user with the VR environment, more practice sessions were performed as compared to the pencil-and-paper versions, which might introduce learning effects.

As detailed in Additional file 1: Table S1*,* the 147 participants in this study performed the CTT tasks while participating in different larger protocols. This could potentially affect, e.g., the test–retest reliability results. However, our post-hoc analyses did not show substantial effect (see *Results* & Additional file 1).

Some limitations are related to the VR media used. For example, visual acuity of the participant is more critical for performance of the HMD-CTT versions than for the pencil-and-paper version, in which the paper remains at a constant, comfortable distance at which all potential targets are visible. The 3D HMD-CTT is fundamentally different in this respect, as the potential targets are located at a variety of virtual depths.

### Future directions

VR technologies may enable us to enrich the current VR-based versions of the CTT to further enhance ecological relevance, mainly in the sense of engaging more modalities, and inter-modalities interactions.

The challenge will then be how to leverage multimodal measures to understand such real-world processes as cognitive-motor interference during multi-tasking and ultimately assess function in a predictive or clinically meaningful way. In particular, we hope to achieve superior discriminant validity for patient cohorts and the ability to predict risks associated with impaired cognitive-motor interactions [58–61], such as the risk of falls in the elderly and in neurological patients [62, 63].

Finally, we envision developing adaptations of additional neuropsychological tests, with different core construct (i.e., than the CTT) for application in an immersive VR environment.

## Conclusions

In sum, the present study describes the development and validation of large-scale (DOME-CTT) and head-mount (HMD-CTT) VR adaptations of the classic pencil-and-paper Color Trails Test (CTT) and provides key validation data, including construct validity relative to the original test, discriminant validity among age groups, and test–retest reliability at two different retest intervals. Critically, this work demonstrates the feasibility and viability of converting a neuropsychological test from two-dimensional pencil-and-paper to three-dimensional VR based format while preserving core features of the task and assessing the same cognitive functions. Our novel findings on the relationship between classical cognitive performance and upper-limb motor planning and execution may lead to new analysis methods for other more ecological VR-based neuropsychological tests that incorporate cognitive-motor interactions.

## Supplementary Information

**Additional file 1:** Additional information on Methods and Results.

**Additional file 2:** Video demo of DOME-CTT.

**Additional file 3:** Video demo of HMD-CTT.

## Data Availability

The datasets used and/or analysed during the current study are available from the corresponding author on reasonable request.

## Abbreviations

ANOVA: Analysis of variance
AUC: Area under the curve
CTT: Color trails test
FOV: Field of view
HMD: Head mounted device
ICC: Intraclass correlation coefficients
MA: Middle-aged adults
MET: Multiple errands test
ROC: Receiver operating characteristic
SD: Standard deviation
TMT: Trail making test
VET: Virtual errands test
VR: Virtual reality
YA: Young adults
